# Real-world clinical effectiveness and sustainability of universal bloodborne virus testing in an urban emergency department in the UK

**DOI:** 10.1038/s41598-022-23602-1

**Published:** 2022-11-10

**Authors:** Elizabeth Smout, Khine Phyu, Gareth J. Hughes, Lee Parker, Roozbeh Rezai, Amy Evans, Joscelyne McLaren, Stephen Bush, Sarah Davey, Mark A. Aldersley, Murad Ruf, Emma E. Page

**Affiliations:** 1 UK Field Epidemiology Training Programme, UK Health Security Agency, Leeds, UK; 2 Field Service, UK Health Security Agency, Leeds, UK; 3 Leeds Teaching Hospitals Trust, Leeds General Infirmary, Great George St, Leeds, LS1 3EX UK; 4 Public Health, Medical Affairs, Gilead Sciences Ltd, London, UK

**Keywords:** Diseases, Gastroenterology, Health care, Risk factors

## Abstract

Innovative testing approaches and care pathways are required to meet HIV, hepatitis B (HBV) and hepatitis C (HCV) elimination goals. Routine testing for blood-borne viruses (BBVs) within emergency departments (EDs) is suggested by the European Centre for Disease Prevention and Control but there is a paucity of supporting evidence. We evaluated the introduction of routine BBV testing in EDs at a large teaching hospital in northern England. In October 2018, we modified the electronic laboratory ordering system to reflex opt-out HIV, HBV and HCV testing for all ED attendees aged 16–65 years who had a routine blood test for urea and electrolytes (U&Es). Linkage to care (LTC) was attempted for newly diagnosed patients, those never referred and those who had previously disengaged from care. The project operated for 18 months, here we present evaluation of the initial nine months (2 October 2018–1 July 2019). We analysed testing uptake, BBV seropositivity, LTC and treatment initiation within six months post-diagnosis. Over 9 months, 17,026/28,178 (60.4%) ED attendees who had U&Es performed were tested for ≥ 1 BBV. 299 active BBV infections were identified: 70 HIV Ab/Ag-positive (0.4% seroprevalence), 73 HBsAg-positive (0.4%) and 156 HCV RNA-positive (1.0%). Only 24.3% (17/70) HIV Ab/Ag-positive individuals required LTC, compared to 94.9% (148/156) HCV RNA-positive and 53.4% (39/73) HBsAg-positive individuals. LTC was successful in 94.1% (16/17) HIV Ab/Ag-positive and 69.3% (27/39) HBsAg-positive individuals. However, at 6 months LTC was just 39.2% (58/148) for HCV RNA-positive individuals, with 64% (37/58) of these commencing treatment. Universal opt-out ED BBV testing proved feasible and effective in identifying active BBV infections, especially among marginalised populations with reduced healthcare access. Our integrated approach achieved good LTC rates although further service development is necessary, particularly for HCV RNA-positive people who inject drugs.

## Introduction

HIV, hepatitis B (HBV) and hepatitis C (HCV) are major global public health threats. Late and undiagnosed infections with these bloodborne viruses (BBVs) cause significant and predictable morbidity and mortality, placing a substantial burden on both healthcare services and society. Global health elimination targets exist for all three BBVs^1,2^. To achieve these goals, innovative approaches are required for identifying undiagnosed infections and improving access and engagement with care. Effective emergency department (ED) BBV testing has been described in the literature and in recent guidance from the European Centre for Disease Prevention and Control (ECDC)^3–10^.

In the UK, while national guidelines recommend routine ED HIV testing in high prevalence areas (≥ 2 per 1000)^11^, implementation is not consistent, especially outside of London. There is emerging UK and international evidence to suggest that systematic ED testing and linkage to care (LTC) for HBV and HCV is effective^5,8,9,12^ and, in the UK, is likely to be highly cost-effective at relatively modest seroprevalences^13^. Current UK hepatitis testing guidelines largely only recommend traditional risk-based testing, but the latest review highlighted that ED testing would be examined as an area of interest^14^. Leeds, a city in northern England, has higher rates of HIV, HBV and HCV than other areas of the UK but far lower rates than seen in London^15–17^.

We evaluated effectiveness and sustainability of an innovative real-world service model, modifying the ED electronic laboratory requesting system to include universal opt-out BBV testing combined with novel integrated care pathways. The project operated for 18 months, pausing due to the COVID-19 pandemic. Here we present evaluation of the initial nine months (2 October 2018–1 July 2019).

## Methods

### Study population and design

We performed a service evaluation of routine opt-out testing for HIV, HBV and HCV and an integrated treatment pathway among ED patients at Leeds Teaching Hospitals NHS Trust (LTHT), Leeds, UK. This evaluation covers the first nine months from implementation on 2 October 2018 to 1 July 2019. We modified the ED electronic laboratory test ordering system so that HIV, HBV and HCV tests were pre-selected with any blood order for urea and electrolytes (U&E) in adult patients (aged ≥ 16 and ≤ 65 years). The lower age cut-off of 16 years was used as this is the age at which a young person in England is presumed to have the capacity to consent. 65 was used as the upper age limit as this had been used in a local acute medical admissions HIV testing pilot and previous projects had demonstrated that positivity rates drop off significantly after the 7th decade^18,19^. Testing uptake was measured for each individual BBV.

Patient information leaflets, describing the testing policy in the six most common local languages, were distributed on registration. Posters were also displayed throughout the ED. Adoption of a notional consent policy was agreed with LTHT Risk Management (responsible for assessing, managing and mitigating risks to patients and staff) whereby patients undergoing blood tests were made aware via posters or multilingual information leaflets that they would be tested for BBVs unless they opted out at the time of having their blood drawn. No verbal consent process was required. This was agreed to minimise operational barriers to testing in the ED setting. Testing was not performed on any patients who declined or who were assessed not to have capacity to consent by the standard capacity assessment used by the healthcare professional managing their current presentation.

An attempt was made to reduce unnecessary testing of patients who had BBV testing within the last 6 months or were already in care for a BBV. Upon electronic request of U&Es, the ED electronic laboratory test ordering system performed an automated lookback for any previous BBV results for the requesting clinician to review. ED clinicians could choose not to test for one or all BBVs if they felt these were not required or would be unnecessary. Unfortunately, this did not prevent all unnecessary testing.

### Laboratory analysis

Serum samples were screened for HIV antibody/antigen, HBV surface antigen (HBsAg) and HCV antibody via ADVIA-Centaur-CP assay (Siemens Healthineers). Any reactive serology results had confirmatory testing via the LIAISON® XL (DiaSorin). Samples reactive on first- and second-line HIV antibody/antigen assays were tested using the Geenius (Bio-Rad Laboratories) to differentiate between HIV-1 and HIV-2. Confirmed positive HBsAg samples were tested for core antibody, e-antigen and e-antibody. Confirmed positive HCV antibody samples were reflex tested for HCV RNA on the Alinity M (Abbott Molecular) to be able to differentiate between those with past and current infection.

### Linkage to care pathways

Upon identification of a confirmed positive patient (HIV Ab/Ag-positive, HBsAg-positive or HCV RNA-positive), an automated secure email was sent from the laboratory to either the HIV or viral hepatitis specialist nurses who checked the care status of the patient as either: requiring linkage to care or currently in care. Those requiring linkage to care were made up of 3 different groups: new diagnoses, those known to specialist services but not in care and those previously diagnosed but not known to specialist services. If the patient was identified as requiring linkage to care, already established healthcare pathways were followed. If the individual was still an inpatient, a specialist nurse would perform an initial in-hospital assessment to discuss their diagnosis and treatment options. If the patient had been discharged, they would be contacted by post and offered an appointment in the relevant specialist service. If they did not attend the appointment they were contacted again via phone or letter and offered a second appointment. Failure to attend this second appointment initiated a letter to the general practitioner (GP) to inform them of their patient’s status, encouraging them to refer the patient for specialist care following a discussion with the patient. Importantly, where the patient had no fixed abode, the specialist nurse team attempted to locate and contact the patient through: the local specialist homeless General Practice, local drug and alcohol services and the city’s primary homeless shelter.

Only those patients identified as requiring LTC were included in this part of the analysis. Patients were defined as linked to care if they had a face-to-face consultation with a specialist nurse or clinician within six months of being diagnosed through ED testing. Those not linked to care within 6 months remained on an active list allowing on-going attempts at engagement. Specialist treatment and management of all BBVs was provided according to national guidelines^20–22^.

### Data collection

Data on patient demographics and ED attendance was collected through the routine hospital electronic patient record (EPR). Data on the number of adult ED attendees who had a blood test for U&Es, together with BBV testing data, was extracted from the hospital laboratory information management system (LIMS) on a weekly basis. LTC data, including risk factor information, was recorded by the HIV and viral hepatitis specialist nurses.

### Data analysis

EPR, LIMS and specialist nurse datasets were linked using the patient’s hospital number as a unique identifier. Hospital number was used to de-duplicate ED attendances, U&E and BBV tests. Where a patient had multiple ED attendances, the earliest attendance was retained. For individuals who had multiple BBV tests performed, the record with a reactive BBV test result was retained. This record was appended with additional information from subsequent test records to improve data completeness, where required.

BBV testing uptake and crude seroprevalence rates were calculated; the denominator used was the total number of patients aged 16–65 years who received a blood test for U&Es in the ED during the study period. The distribution of demographic characteristics, reported risk factors and care status among those testing positive for BBVs were described using numbers and proportions with 95% confidence intervals. Ages were categorised in fifteen-year age bands and ethnicity data grouped according to Office for National Statistics (ONS) categories^23^. LTC estimates were calculated through the proportion of patients requiring LTC and successfully linked. Chi-squared tests were used to compare LTC rates between the different BBVs and to estimate variables associated with LTC.

Data cleaning, management and analysis was carried out in R v3.5.2.^24^.

### Ethical approval and consent to participate

This project was a service evaluation according to the NHS Health Research Authority, confirmed by the LTHT Research & Development department by email on 17 July 2017. This is because the project was looking to develop and expand on existing practice which was already in place, and therefore did not require further ethical review by an NHS Research Ethics Committee. Guidance around defining research is available from the NHS Health Research Authority and was used to support this decision (http://www.hra-decisiontools.org.uk/research/docs/DefiningResearchTable_Oct2017-1.pdf). All methods were performed in accordance with relevant guidelines and regulations.

Adoption of a notional consent policy was agreed with LTHT Risk Management and approval was given by email on 6 September 2018 from LTHT’s Associate Medical Director for Risk Management to proceed with the testing of capacitous patients. Posters and information leaflets, available in a variety of languages, were distributed to patients on arrival in the ED, informing patients undergoing blood tests that they would be tested for BBVs unless they opted out at the time of having their blood drawn. No verbal consent process was required. Testing was not performed on any patient who declined or who was assessed not to have capacity to consent by the health care professional managing the current presentation.

## Results

**Figure 1:**
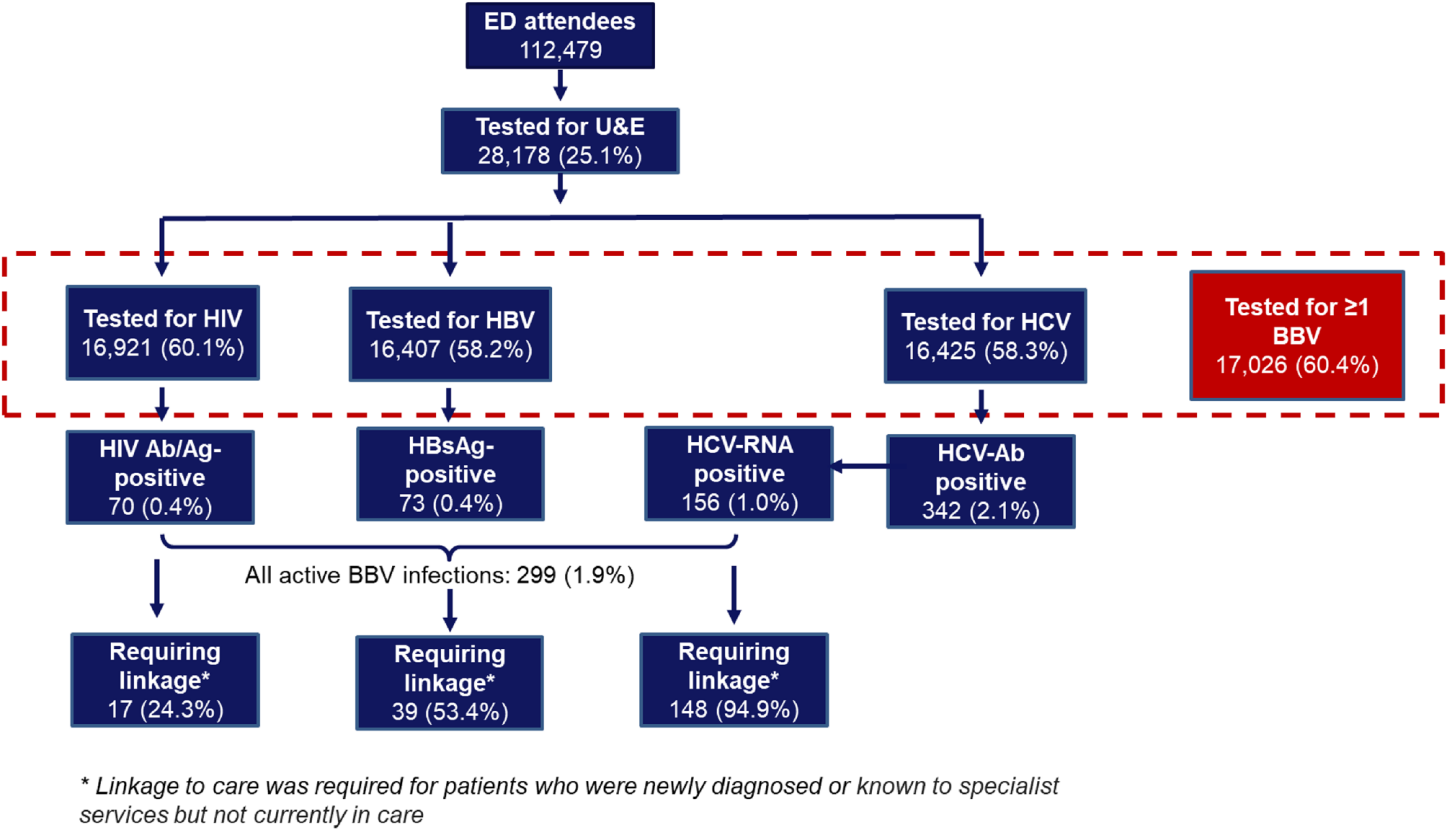
Flow of patients through the BBV testing pathway within the ED testing programme.

The numbers of patients tested for BBVs during the initial nine months of this testing pathway are displayed in Fig. 1. Testing uptake for ≥ 1 BBV was 60.4%. 60.1% of eligible patients were tested for HIV, 58.2% were tested for HBV and 58.3% were tested for HCV.

### Detection of active infections

HIV Ab/Ag prevalence was 0.41% (95% CI 0.32–0.52), HBsAg prevalence was 0.44% (95% CI 0.36–0.57) and HCV RNA-positive prevalence was 0.95% (95% CI 0.80 – 1.10). Coinfection prevalence was low (HIV/HBV: 0.02% (n = 3); HIV/HCV 0.01% (n = 2); HBV/HCV: 0.01% (n = 1)). For all three BBVs, prevalence was higher in males and among individuals aged between 30 and 49 years (Table 1). BBV prevalence was significantly higher in males than females for viral hepatitis, but not HIV. The ethnicity profiles varied considerably between different BBVs but these differences were not significant, except between HCV RNA-positive and HIV Ab/Ag positive (*p* = < 0.01 patients). While the vast majority of HCV RNA-positive patients were White British, the majority of diagnosed HIV and HBV infections were from minority ethnic groups.

**Table 1:** Demographic characteristics of patients testing positive for active BBV infection in the ED.

	BBV					
Demographic characteristic	HIV (n = 70)		HBV (n = 73)		HCV-RNA (n = 156)	
	n	%* (95% CI)	n	%* (95% CI)	n	%* (95% CI)
**Gender**						
Male	40	57.1 (45.1–68.4)	51	69.9 (58.1–79.5)	116	74.4 (66.8–80.7)
Female	30	42.9 (31.6–54.9)	22	30.1 (20.5–41.9)	40	25.6 (19.3–33.2)
**Age (years)**						
16–29	7	10.0 (4.8–19.8)	7	9.6 (4.6–19.1)	13	8.3 (4.9–13.9)
30–49	38	54.3 (42.3–65.8)	48	65.8 (53.9–75.9)	114	73.1 (65.5–79.5)
50–65	25	35.7 (25.2–47.8)	18	24.6 (15.9–36.1)	28	17.9 (12.6–24.9)
Unknown					1	0.6 (0.1–4.5)
**Ethnicity**						
White British	29	41.4 (30.3–53.5)	1	1.4 (0.2–9.5)	137	87.8 (81.6–92.1)
White other (incl. Irish)	6	8.6 (3.8–18.1)	29	39.7 (29.0–51.6)	15	9.6 (5.8–15.4)
Asian British/Asian other	2	2.9 (0.7–11.1)	15	20.5 (12.6–31.6)	1	0.6 (0.1–4.5)
Black British/Black other	32	45.7 (34.2–57.7)	24	32.9 (22.9–44.7)	2	1.3 (0.3–5.1)
Mixed or other	1	1.4 (0.2–9.9)	4	5.5 (2.0–14.0)	1	0.6 (0.1–4.5)

*Percentage of individuals within each category calculated using the denominator for each BBV included in the column header.

### Linkage to care

There was a statistically significant difference between the proportion of patients requiring LTC across all three BBVs (*p* < 0.01) and when comparing each combination of BBV separately (*p* < 0.01 for each of HIV vs. HCV, HIV vs. HBV, HBV vs. HCV). Almost all patients testing positive for HCV-RNA required LTC (94.9%, 95% CI 90.0–97.4%), compared to approximately one half of HBsAg-positive patients (53.4%, 95% CI 41.7–64.8%) and only one quarter of HIV Ab/Ag positive patients (24.3%, 95% CI 15.5–36.0%) (Table 2). 46.2% (95% CI 38.4–54.1%) of HCV RNA-positive patients who required LTC had been known to specialist services but were not currently in care compared to fewer than one-third of HIV Ab/Ag or HBsAg-positive patients. Eleven (7.0%, 95% CI 3.9–12.4%) HCV RNA-positive patients had previously been diagnosed with HCV but were not known to specialist services.

**Table 2:** Care status of BBV-positive patients at the time of diagnosis.

Care Status	BBV					
	HIV (n = 70)		HBV (n = 73)		HCV-RNA (n = 156)	
	n	% (95% CI)	n	% (95% CI)	n	% (95% CI)
**Requiring linkage**	17	24.3 (15.5–36.0)	39	53.4 (41.7–64.8)	148	94.9 (90.0–97.4)
New diagnosis	12	17.1 (9.9–28.1)	35	47.9 (36.5–59.6)	65	41.7 (34.1–49.6)
Known to specialist services but not currently in care	5	7.1 (2.9–16.3)	4	5.5 (2.0 – 14.0)	72	46.2 (38.4–54.1)
Previously diagnosed but not known to specialist services	0	–	0	–	11	7.0 (3.9–12.4)
**Currently in care**	53	75.7 (64.0–84.5)	34	46.6 (35.2–58.3)	8	5.1 (2.6–10.0)
Total	70		73		156	

#### HIV

The majority of HIV Ab/Ag positive patients requiring LTC were male (64.7%, 95% CI 37.7–84.8%, n = 11), with a median age of 46 (range: 16–64 years). Eight (47.1%, 95% CI 23.5–72.0%) were White British, 6 (35.3%, 95% CI 15.2–62.3%) were Black British or Black other and 3 (17.6%, 95% CI 5.1–46.3%) were White other (including Irish). Neither age, sex nor ethnicity were crudely associated with requiring LTC. The median CD4 count for patients requiring LTC was 99 (range 1–593) and more than two-thirds of these patients (64.7%, n = 11) were diagnosed with advanced disease (CD4 cell count < 200 cells/mm^3^)^25^. Just under two-thirds of patients requiring LTC were thought to have acquired HIV through heterosexual sex (58.8%, 95% CI 32.7–80.7%, n = 10), with approximately one-third occurring in men who have sex with men (35.3%, 95% CI 15.2–62.3%, n = 6).

#### HBV

Of HBsAg-positive patients requiring LTC, 71.8% (95% CI 55.1–84.1%, n = 28) were male and the median age was 39 (range 19–63 years). Just 2.6% (95% CI 0.3–17.4%, n = 1) were White British. Approximately half (48.7%, 95% CI 33.0–64.7%,n = 19) of these individuals were White other (including Irish), 30.8% (95% CI 17.9–47.5%, n = 12) were Black British or Black other, 10.3% (95% CI 3.7–25.2%, n = 4) were Mixed or any other ethnicity, 7.7% (95% CI 2.4–22.2%, n = 3) were Asian British or Asian other. Again, neither age, sex nor ethnicity were crudely associated with requiring LTC (all *p* > 0.05). Most HBsAg-positive patients requiring LTC were from an area of high prevalence (HBsAg prevalence of more than 8%^26^) (87.2%, 95% CI 71.8–94.8%, n = 34).

#### HCV

As with HIV and HBV, the majority of HCV RNA-positive patients requiring LTC were male (73.6%, 95% CI 65.9–80.2%, n = 109), with a median age of 41 (interquartile range (IQR) = 35—48). In contrast to HIV and HBV, 87.8% (95% CI 81.4–92.2%, n = 130) were White British, 9.4% (95% CI 5.6–15.4%, n = 14) were White other (including Irish), 1.4% (95% CI 0.3–5.3%, n = 2) were Black British or Black other, 0.7% (95% CI 0.1–4.7%, n = 1) were of Mixed ethnicity and a further 0.7% (95% CI 0.1–4.7%, n = 1) were Asian British or Asian other. As with the other BBVs, neither age, sex or ethnicity were crudely associated with requiring LTC (all *p* > 0.05). The majority of RNA-positive patients were a current person who inject drugs (PWIDs) (56.8%, 95% CI 48.6–64.6%, n = 84), while a further quarter had previously injected drugs (25.7%, 95% CI 19.2–33.4%, n = 38).

### Linkage to care pathways

#### HIV

**Figure 2:**
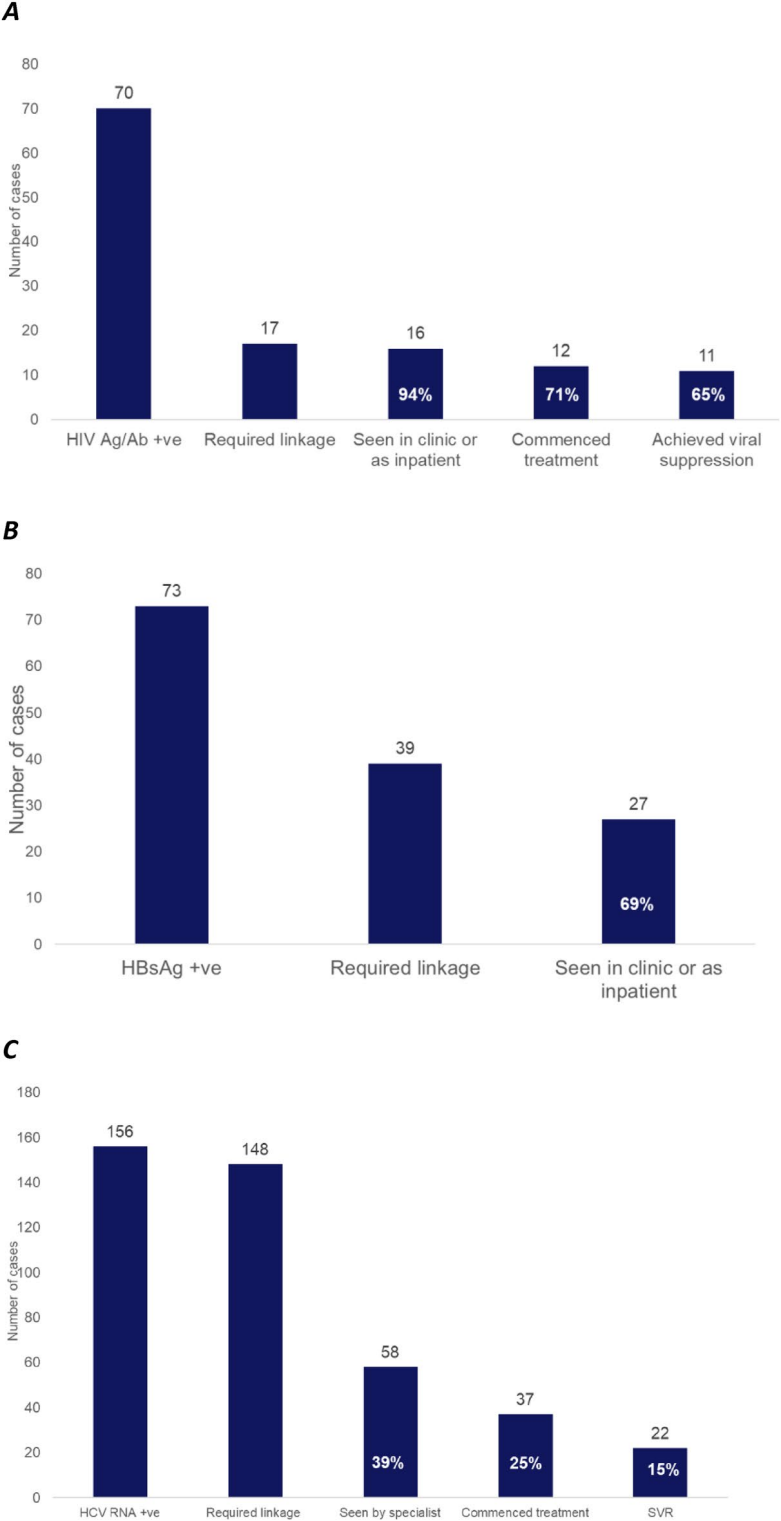
The care continuum within six months following diagnosis of (**A**) HIV infection, (**B**) HBV infection, and (C) HCV infection in the ED. Percentages shown use the number of patients requiring linkage to care as the denominator.

16/17 (94.1%, 95% CI 62.7–99.3%) HIV Ab/Ag positive patients were successfully linked to care (Fig. 2A). 5/17 HIV Ab/Ag positive patients diagnosed within the ED (29.4%, 95% CI 11.5–57.1%) were known to specialist services but not in care at the time of their ED visit; four of these five patients (80.0%, 95% CI 11.1–99.21%) successfully linked to care. All those newly diagnosed in ED were linked to care.

Twelve (70.6%, 95% CI 42.9–88.5%) patients linked to care commenced antiretroviral treatment (ARVs) within six months; of the remaining four individuals, three were known to specialist services but had previously disengaged. Eleven patients (64.7%, 95 CI 37.7–84.8%) achieved viral suppression (HIV viral load < 200 copies/ml) within six months on treatment. One patient died before completing six months on ARVs.

#### HBV

27 of 39 HBsAg-positive patients (69.2%, 95% CI 52.5–82.1%) were successfully linked to care (Fig. 2B). Of the twelve patients not linked, four (33.3%, 95% CI 10.9–67.1%) lived out of area, seven (58.3%, 95% CI 26.7–84.3%) were newly diagnosed with HBV for the first time within the ED and one (8.3%, 95% CI 0.8–50.1%) was known to specialist services but had previously disengaged. Three of the 27 individuals (11.1%, 95% CI 3.3–31.1%) who were successfully linked to care had previously been linked to care but had disengaged by the time of their ED attendance, and were therefore re-engaged because of their ED testing.

#### HCV

Only 58 of 148 patients requiring LTC (39.2%, 95% CI 31.6–47.4%) were seen in clinic or as an inpatient (Fig. 2C). In total, one quarter (25.0%, 95% CI 18.6–32.7%, n = 37) of HCV RNA-positive patients requiring LTC commenced treatment within six months following ED diagnosis. LTC rates were lower for current PWIDs compared to those who did not currently inject drugs (*X*_2 (1,148)_ = 4.15, *p* = 0.04). The only factor crudely associated with commencing treatment was PWID status, with current PWIDs being significantly less likely to start treatment than those not currently not using drugs (odds ratio = 0.11, 95% CI 0.03–0.36, *p* < 0.001).

Two-thirds (64%, n = 58) of those patients who did not engage with treatment within six months were current PWIDs. In contrast, current PWIDs were underrepresented within the population who successfully commenced treatment, with only 29.7% (95% CI 16.8–47.0%) of those starting treatment being current PWIDs despite 56.8% (95% CI 48.6–64.6%) of those requiring LTC having this risk factor for infection. A further 18 patients were LTC by 12 months following diagnosis, with 4 of these patients starting treatment.

Of the 37 patients who commenced treatment for active HCV within six months of their ED diagnosis, 22 (59.5%, 95% CI 42.4–74.5%) had a sustained viral response (SVR) at 12 weeks post-treatment. Just two patients (5.4%, 95% CI 1.3–20.3%) were considered to have experienced true treatment failure. The other thirteen patients (35.1%, 95% CI 21.1–52.4%) were either lost to follow-up (n = 11) or failed to comply with treatment (n = 2).

### Mortality

Nine BBV-positive patients died during the evaluation period: seven HCV RNA-positive and two HIV Ab/Ag positive patients, all of whom required LTC. Both HIV Ab/Ag positive patients died from HIV-related complications and were new diagnoses with CD4 < 100 at the time of their ED visit. The cause of death for all six HCV RNA-positive patients was not related to their HCV diagnosis.

## Discussion

This real-world service evaluation demonstrates the feasibility, effectiveness and sustainability of routinely testing for BBVs within the ED, in line with guidance from the British HIV Association (BHIVA) and ECDC^27,28^. This is the first evaluation to-date outside of extremely high prevalence London, with a large number of active BBV infections identified requiring LTC, providing evidence for the effectiveness of routine ED testing outside ‘traditional hotspot’ areas.

We achieved a higher testing uptake than seen in previous studies^3–5^, with this high testing rate continuing throughout the full 18-month period. This intervention was successfully continued for a further nine months (until 31 March 2020) when it had to be stopped (by switching off the reflex BBV testing attached to U&Es in the electronic laboratory ordering system) due to NHS England guidance on the prioritisation of laboratory testing during COVID. During the 2nd nine-month period between 1 July 2019 and 31 March 2020, BBV testing uptake within the ED remained high and was similar to testing uptake rates seen during the initial nine-month testing period (as described in Fig. 1): 51.5% for HIV (16,905/32,827), 49.7% for HBV (16,331/32,827) and 49.7% for HCV (16,318/32,827)^29^.

In the six months following ‘switch-off’ (1 April–30 September 2020), BBV testing uptake within the ED dropped to 0.7% for HIV (89/13,211), 0.5% for HBV (70/13,211) and 0.5% for HCV (71/13,211). These testing uptake rates are similar to those seen in the 6 months prior to the ‘switch-on’ of the reflex BBV testing attached to U&Es in the electronic laboratory ordering system (1 April–1 October 2018): 0.4% for HIV (95/21,953), 0.3% for HBV (62/21,953) and 0.3% for HCV (67/21,953).

As demonstrated above, the key to the effectiveness and sustainability of our BBV testing model was the automation of test requesting through use of an electronic laboratory ordering system. This reflex requested BBV tests when U&Es were requested, instead of using a more traditional nurse-led model. This, together with the universal notional consent policy, normalised routine testing for BBVs within the ED. A recent systematic review has shown that universal testing approaches reduce barriers to testing that may be caused by stigma, with patients feeling less singled out and staff feeling more comfortable to offer BBV testing^30^. This approach also minimises the impact of introducing routine testing on frontline ED staff working in a busy setting.

Our results from an urban centre in northern England are compatible with other largely London-based ED testing studies (HIV Ab/Ag seroprevalence: 0.2–1.2%^4,8,9,31^, HBsAg: 0.5–2.0%^4,5,8,9,12,13^, and HCV-RNA: 1.2–2.9%)^3–5,8,9,12,13,32^. Although there is variation likely related to differences in the local populations, seroprevalences identified through ED testing are consistently far higher than estimated general population prevalence^16,33^. This is likely because EDs tend to provide access to healthcare to individuals who do not routinely engage with other health services and who may be at higher risk of BBVs. Rates of substance misuse are higher among ED attendees than the general population^34^, while estimates suggest that homeless people use ED services up to seven times more often than the general population in England^35^.

We found striking differences in LTC outcomes for HIV, HBV and HCV patients. There is very little published data regarding real-world LTC outcomes following testing in either EDs or other settings outside of controlled studies, but the proportion of patients requiring LTC for active HCV infection was consistently high across all studies^4–9^, and was notably higher than for HBsAg- or HIV Ab/Ag positive patients. Similarly, just under half of HCV patients who required LTC were known to specialist services but were not in care at the time of their ED visit, compared to fewer than one-third of HIV Ab/Ag or HBsAg-positive patients. This is probably explained by the difference in at-risk populations and risk factor profiles between the BBVs, especially pronounced for HCV, with most patients being current PWIDs.

We found the majority (64.7%) of HIV Ab/Ag positive patients requiring LTC were diagnosed with very late stage infection (CD4 < 200) and heterosexual (58.8%). This may result from the stigma that still surrounds HIV infection, as well as risk perception among heterosexual people compared to men who have sex with men, although we are unable to assess whether LTC following HIV diagnosis was associated with route of acquisition as sexual orientation was not available for those individuals who did not require LTC. These results suggest that introducing universal opt-out ED testing can provide a safety net for those patients at risk of HIV and who are less likely to engage with other healthcare services. Furthermore, routine ED testing may have the potential to reduce the proportion of late HIV infections diagnosed within the ED as reflected in a key recommendation in the recent UK HIV Commission report^36^.

Despite high testing rates and a service model that combined ED testing with an integrated care pathway, the findings of this service evaluation illustrate that while LTC for HIV and HBV was good, a challenge remains in successfully linking HCV RNA-positive current PWID patients. Patients actively using drugs were significantly less likely to be successfully linked to specialist care, despite network LTC efforts involving outreach into local drug services, homeless shelters and via the specialist GP for the homeless. This demonstrates the need to continue to change the way in which HCV RNA-positive patients are identified and offered treatment, requiring continued service network development involving community services, and echoing the findings of a recent systematic review from the UK Health Security Agency^30^. Our findings also suggest that if patients did not engage with care and begin treatment within the first 6 months following diagnosis, they were unlikely to be successfully linked in the future. This is likely explained by the underrepresentation of current PWIDs among patients successfully commencing treatment compared to those requiring LTC; this indicates that the first patients to be linked to care are likely to be the easiest to engage, while successfully commencing current PWIDs on treatment is more difficult to achieve.

Over recent years, there has been a gradual shift in the focus of more patient-centred treatment, away from hospitals and into appropriate community settings such as drug services and homeless services. Nevertheless, further innovation is needed to ensure the current elimination goals are met through initiatives such as embedding clinics within drug services, scaling up the use of peer mentors, digital adherence solutions and the use of simplified treatment regimens.

### Limitations

There are several limitations to this real-world service evaluation. Due to inaccuracies in data reporting we were unable to remove individuals from the denominator who were ineligible due to them not having the capacity to consent, having previously tested positive or having tested in the last 6 months, meaning that BBV uptake will have been underestimated. Similarly, we were unable to collect detailed information regarding the profile and reasons of those who declined testing; an attempt was made to capture this information, but an initial review of this data demonstrated that reasons for declining testing were not accurately recorded by the ED staff. We therefore cannot be certain that those who were tested as part of this evaluation were representative of the population attending the ED. Finally, BBV testing was not performed on those who did not have capacity to consent e.g. patients who were intoxicated. This may have resulted in this intervention missing some of those most at risk of testing positive for a BBV.

## Conclusion

These findings demonstrate the real-world effectiveness and sustainability of our innovative universal, opt-out ED BBV testing pathway at far lower seroprevalence thresholds than stated in the ECDC guidelines^28^. ED testing identified a large number of active BBV infections requiring LTC. We identified a high proportion of late-stage HIV infections and disproportionately diagnosed heterosexuals. Both the need for and challenge with LTC was a particular issue with HCV patients, particularly those who were currently injecting drugs. These findings demonstrate the need to continue to evolve the current service model, focusing efforts on supporting patients to commence and complete treatment within the community. A dedicated cost-effectiveness analysis using data from this study is in progress, to inform UK guideline development and implementation.

## Data Availability

The datasets generated during this service evaluation are available on request. All enquiries about the data used for this evaluation should be directed to the corresponding author.

## Abbreviations

HIV: Human Immunodeficiency Virus
HBV: Hepatitis B virus
HCV: Hepatitis C virus
BBV: Blood-borne virus
ED: Emergency department
ECDC: European Centre for Disease Control and Prevention
LTC: Linkage to care
LTHT: Leeds Teaching Hospitals NHS Trust
U&E: Urea & Electrolytes
EPR: Electronic patient record
LIMS: Laboratory information management system
PWID: People who injects drugs
ARVs: Antiretrovirals
BHIVA: British HIV Association
